# THE EFFECTS OF HOUSING AND LIVING STANDARDS ON LONELINESS AND SOCIAL EXCLUSION AMONG OLDER PEOPLE

**DOI:** 10.1093/geroni/igac059.1050

**Published:** 2022-12-20

**Authors:** Charles Waldegrave, Chris Cunningham, Catherine Love, Giang Nguyen

**Affiliations:** Family Centre Social Policy Research Unit, Lower Hutt, Wellington, New Zealand; Research Centre for Maori Health and Development, Wellington, Wellington, New Zealand; Family Centre Social Policy Research Unit, Wellington, Wellington, New Zealand; Family Centre Social Policy Research Unit, Wellington, Wellington, New Zealand

## Abstract

**Introduction:**

Housing security is a likely indicator of loneliness (Gierveld et. al. 2015, Gonyea et. al. 2018) but there are few studies that focus specifically on the relationship between the two. This paper presents findings from a New Zealand Ageing Well National Science Challenge research programme that presents findings on the impacts of housing and living standards on loneliness and social exclusion. Method: This presentation will provide results from a study of 200+ Māori (indigenous New Zealanders) aged 50 years and over. Key questions around loneliness and social isolation were co-created with the participants and responses compared with standard international scales to help identify both universal aspects of loneliness and culturally specific aspects. Questions relating to housing security, affordability, living standards and neighbourhood safety were also asked. Regression analysis was used to test the statistical significance of the various relationships between differing aspects of housing and loneliness.

**Results:**

The results demonstrate statistically significant relationships between housing quality, affordability, living standards and neighbourhood suitability with both the universal and culturally specific scales of loneliness. Overall greater housing security and quality was shown to be negatively associated with loneliness, which suggests it contributes to the reduction of loneliness.

**Conclusion:**

Addressing the quality of social connections has often been seen as the key way to address loneliness. The results of this study suggest housing security and living standards play an important role in people’s perception of loneliness and their experience of it, as well.

